# A Lightweight Neighbor-Info-Based Routing Protocol for No-Base-Station Taxi-Call System

**DOI:** 10.1155/2014/601913

**Published:** 2014-03-09

**Authors:** Xudong Zhu, Jinhang Wang, Yunchao Chen

**Affiliations:** School of Computer Science & Information Engineering, Zhejiang Gongshang University, Hangzhou 310018, China

## Abstract

Since the quick topology change and short connection duration, the VANET has had unstable routing and wireless signal quality. This paper proposes a kind of lightweight routing protocol-LNIB for call system without base station, which is applicable to the urban taxis. LNIB maintains and predicts neighbor information dynamically, thus finding the reliable path between the source and the target. This paper describes the protocol in detail and evaluates the performance of this protocol by simulating under different nodes density and speed. The result of evaluation shows that the performance of LNIB is better than AODV which is a classic protocol in taxi-call scene.

## 1. Introduction 

The VANET has characteristics of mobile ad hoc network, such as self-organization, flexible structure, multihop routing, dynamic changes of network topology, and need of good scalability. Special application environment, such as narrow road, high density node distribution, high-speed node moving, and other factors, will directly influence the information transmission ability of VANET network, which leads to the increase of packet loss and delay. Meanwhile, the limited wireless channel and huge network scale challenge the data transmission of VANET.

The key to solve the above problems is how to design a routing protocol [1–7]. Since the nodes in VANET are of high mobility, it results in connection failure while applying routing protocol of the traditional MANET [8–10], such as AODV [11–13] and DSR [14, 15], into the VANET, thus leading to delay or even failure to transmit information to the target node.

Due to the special environment in VANET, it cannot have a widely applicable routing algorithm. The typical algorithm in VANET, such as GSR routing protocol, involves each node storing the neighbor lists, topology table, next hop table, and distance table. All of them maintain the state information of adjacent nodes and choose the appropriate router according to the location and topological information. In small network with high mobility and limited bandwidth, the performance of transmission is good However, it requires the node to maintain the network topology. And with the increase of network size, the routing information that needs to be exchanged will increase exponentially. Another type of algorithm, such as the GPSR [16, 17], is based on position. It depends on the overall geographical location information search system. It cannot work without GPS.

When applied to the urban taxi-calling scene, the VANET vehicle network has the following characteristics. (1) The density distribution of vehicle is uneven. Places such as the business center, theaters, and bus stations are dense. The traditional VANET routing protocols have serious information channel congestion problem. (2) The calling passenger has no special requirements to the not-taken taxis, so all the nearby taxis can serve. While the traditional VANET routing protocols always have designated transmission target, or specifies the routing service of all nodes in the target area, which will lead to huge routing overhead, further deteriorating the channel congestion problem. (3) The data transmission is time valid. Passengers' calls need to be responded to in a short time, while the routing connection time is fairly long in some of the traditional VANET routing protocols. Therefore, it cannot effectively find not-taken taxis with such application and will have bad effects on passengers' experiences. We describe some of the work roughly in [18].

This paper aims to solve the quick topology change, short connection duration, and unstable wireless signal quality of the VANET network, thus proposing a kind of lightweight routing protocol applicable to the urban taxi-call without base stations. The protocol maintains and predicts neighbor information dynamically in order to find the reliable path between the source and the target. This paper describes the protocol in detail and evaluates the performance of this protocol by simulating under different nodes density and speed. The result of evaluation shows that the performance of LNIB is better than that of AODV which is a classic protocol in taxi-call scene.

The second chapter analyzes the application and proposes the model; the third chapter describes the routing protocol and its algorithm; the fourth chapter is about the experiment and data analyses; chapter five concerns other related research; the last part will draw a conclusion.

## 2. Transmission Mode and Analyses

In the taxi-calling scene, the vehicle network has two sets: taxi nodes *Q* = {*q*_1_, *q*_2_,…*q*_*n*_} and passenger nodes *P* = {*p*_1_, *p*_2_,…*p*_*m*_}. From the start of the call to get on the car or the call to cancel, a connection should be set up in the network. Because the passenger always waits at the original place or moves slowly, suppose *p* is a static node. And *p* only acts as the initiator of data transmission or receiver, not as mediator in other routings. The node *q* can send, receive or act as a routing node to transmit data. Assume that the speed scalar of each node in *Q* is |*v*|.

When *p* initiates a call, the message transits through the taxi nodes, so *p* can quickly find nearby not-taken cars node *q*_*s*_ that can meet the requirements.

Supposing there are two nodes *a* and *b* in the network, the signal coverage radii for them are, respectively, *R*_*a*_ and *R*_*b*_. If the distance between *a* and *b* is *D*_*a*,*b*_ ≤ min⁡(*R*_*a*_, *R*_*b*_), it can be called “meet.” To be more general, assuming that signal coverage radius of each node in the network is *R*, then “meet” in time *t* means *D*_*a*,*b*_(*t*) ≤ *R*. Define *H*_*a*,*q*_*s*__ as the minimal number of hops to transmit message from *a* to *q*_*s*_. Among all *q*_*s*_ that get the call request, *H*_*a*_ is the lowest of *H*_*a*,*q*_*s*__.

### 2.1. Prediction Based on Neighbor Information

In order to percept the current meet nodes in real time, *q* needs to send broadcast packets to notice their own state information periodically. In the starting time *t* of every broadcast cycle, *q* measures their own motion state, including the speed, the current position, and other information. According to the above information, *q* predicts the coordinates at the time of *t* + *t*_*f*_ and broadcasts the position. The broadcast cycle is *t*_*b*_. If *t*_*b*_ is small, then the channel will be occupied by a large number of broadcast packets, thus influencing the normal data transmission. However, large *t*_*b*_ will lose the prediction value due to the frequent changes in actual situation. Therefore, *t*_*b*_ should be set properly, such as 10 seconds. A broadcast cycle can be divided into multiple correction cycles. The node in a correction period measures their movement state. While finding the coordinates' different Δ*s* between the current forecast coordinate at *t* + *t*_*f*_ and the last broadcast coordinates surpasses, it indicates that the vehicle's driving condition changes. If Δ*s* is greater than a certain value, the node broadcasts the position again to correct the predict position of itself in neighbor nodes' state table, thus to ensure the prediction accuracy. Suppose the correct period is *t*_*r*_.

Meanwhile, if *q* receives the position broadcast of the nearby nodes, it maintains an adjacent link (see Table 1) to record node information that meets with *q*. It can be seen from the table that *q*_2_ measures its position and velocity at 13:11:12′36 and broadcasts. *q*_1_ receives the position broadcast. If *D*_*q*_1_,*q*_2__(*t*) ≤ *R*, then it can be predicted that, at *t* + *t*_*f*_, *q*_1_ and *q*_2_ will keep meeting. If *q*_2_ is not in the table, *q*_1_ adds the information of *q*_2_ to the table and sets *q*_2_ to be active. If *q*_2_ is already in the table, update the information of *q*_2_. When *q*_1_ receives the correction broadcast of *q*_2_, the position information of *q*_2_ also needs updating. If it can be predicted that *q*_1_ and *q*_3_ will not meet at *t* + *t*_*f*_, set *q*_3_ to be inactive. As to the inactive node in the table, if no broadcasts are received in more than one broadcast cycle, then delete the entry of this inactive node. Each time the passenger calls a taxi, several links will be produced. If *q*_1_ and *q*_3_ are neighbors and are both in one link, then they set up link ID in the entry for each other in their own table. Link ID is a large number that is randomly generated. It can be assumed that it is unique in a limited region.

When *q*_1_ receives the broadcast of neighbors at time *t*, it will predict coordinates of itself and neighbor nodes at *t* + *t*_*f*_, respectively. If the two cars can keep communication at that time (such as *q*_2_), the communication is effective. Therefore *q*_1_ stores the information of *q*_2_ in the table otherwise discards it (such as the *q*_3_).

In order to provide the position information and routing of not-taken taxis, the hop distance should be introduced when *q* node is broadcasting message (including position and correction broadcast). Periodically *q*_*s*_ sets *H*_*q*_*s*__ to 0 and sends broadcast. The *q*_*s*_ discards any broadcast information it received without processing. The *q* sets *H*_*q*_ value to ∞, when no neighbor nodes can reach *q*_*s*_ node. When *q*_1_ receives the broadcast of *q*_2_ with the active state, if *H*_*q*_2__ + 1 < *H*_*q*_1__, *q*_1_ modifies the *H*_*q*_1__ value to *H*_*q*_2__ + 1.

Once *H*_*q*_1__ changes, *q*_1_ will spread to notify neighbor nodes to be updated by periodical breakfast. But each cycle can only update 1 hop on the link. If *H*_*q*_1_,*q*_2__ = *n*, it needs *n* cycle to update *H*_*q*_2__.

In order to improve the broadcast speed of *H*, the sending phase of each cycle is divided into two time slices. The node of *H* changes, but no broadcast will be broadcasted at the first time slice. If the *H* value has no change after last broadcast, the node broadcasts in the second time slice. Thus, the value of *H* may spread 2 hops in a cycle. For the length of link will be limited in 3-4 hops in the actual system, two cycles can complete the information disclosure of not-taken taxis by the improved broadcast speed.

### 2.2. Links Setting and Handshake

When *p* launches the call, it needs to find suitable not-taken taxis *q*_*s*_ as soon as possible and make an appointment. In this process, the following questions need to be solved. First, how to find *q*_*s*_ in the shortest time and establish the link to *q*_*s*_? Secondly, if there are several *q*_*s*_ nodes, how to choose one of the most suitable ones to make an appointment?

The simplest method is to use the broadcast to send out the call until a node of *q*_*s*_ receives the request and returns a response. The response will go backtracking to the *p* node. If *p* receives several responses, then choose one from them, after which appointment confirmation will be sent to the chosen node through the path just set up. Meanwhile, it will send negative responses to other not-taken taxis. This method is easy to be implemented, but in the real VANET scene, there are serious problems; the message must pass the whole link three times: request, response, and appointment. On the one hand, it will lead to serious delay and difficulties in keeping the link; on the other hand, the flooding of breakfast will deteriorate the network environment and influence the normal communication.

**Figure 1:**
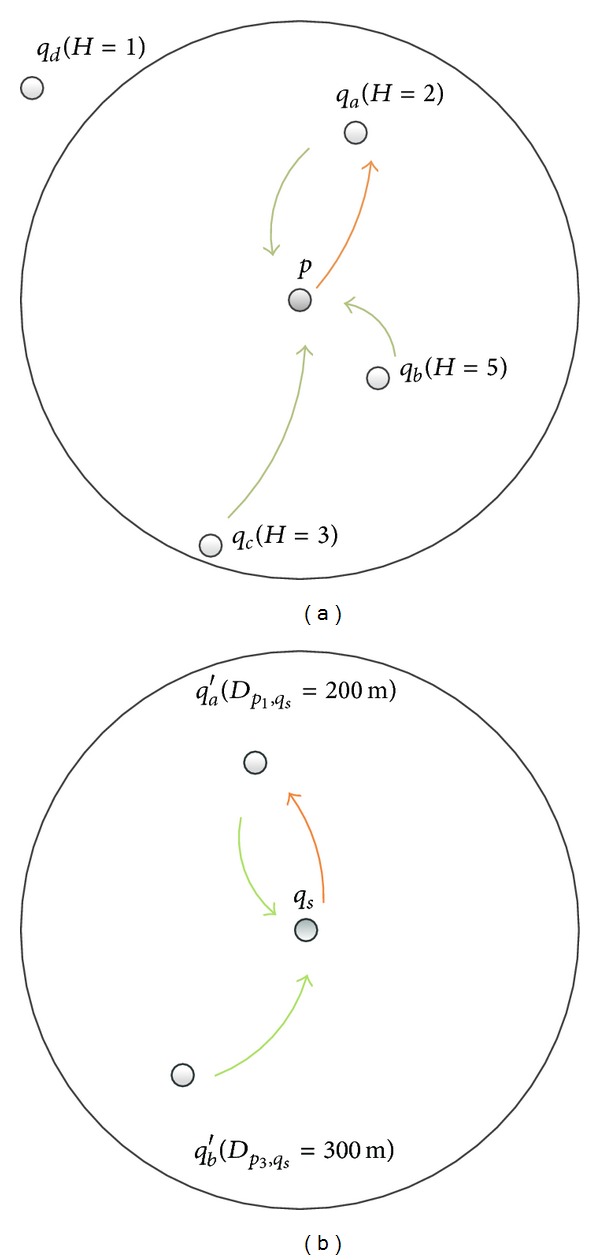
(a) If there are the *q* nodes within the single hop range of *p*, then they shake hands and choose one as *q*_*a*_ that has minimal hop distance to *q*_*s*_ to make an appointment. (b) If there are several *q* nodes around *q*_*s*_ and several *p* nodes, send the appointment requests, then they shake hands and choose the nearest *p* node to response.

**Figure 2:**
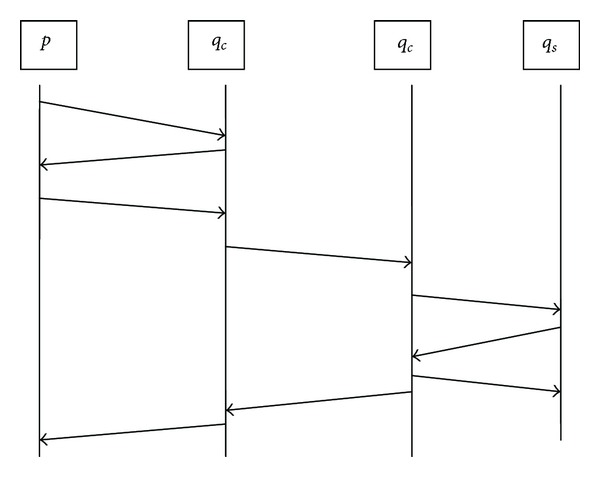
The two-end handshake agreement.

This paper proposes a two-end handshake agreement to great link (show as Figures 1 and 2) According to the neighbor nodes' information described in Section 2.1, each node can know the nearest (by hop distance) *q*_*s*_ and the shortest route to arrive at the node. If *p* sending the request broadcast, its neighbor *q* node which receives *p*'s breakfast directly can judge whether it can reach *q*_*s*_ or not. In fact, it is supposed that the whole data transmission is a single route (the node on the link will transmit a message to only one neighbor node). Once *q* is identified as the first hop, the routing from *p* to *q*_*s*_ will also be established. The *q* can directly send a request response to the *p*. If *p* receives responses from several *q* nodes, it chooses a *q* node as *q*_*a*_ to make appointment confirmation and send a veto appointment to other response *q*. After that *q*_*a*_ accepts the confirmation and sends it to the *q*_*s*_ based on the routing information, that is, to transmit message to the neighbor *q* node in which *H*_*q*_ = *H*_*q*_*a*__ + 1. On the routing, node *q*′ will record the information of last hop, while receiving the message. Meanwhile, transmit to the next hop which *H*_*q*_ = *H*_*q*′_ + 1 until sent to the node which *H*_*q*_ = 0, that is *q*_*s*_.

The *q*_*s*_ end of the link also needs handshaking. If several neighbor *q* nodes, in which *H*_*q*_ = 1, are for the same *q*_*s*_ node, they will send appointment requests to the *q*_*s*_, and the *q*_*s*_ sends a confirmation response to one of them and negative responses to others.

The two-end handshaking method can reduce the three times handshaking to two times, thus reducing the risk of delaying and disconnection. Meanwhile, the broadcast can be controlled in 1 hop and can avoid the flooding.

### 2.3. Disconnection

Suppose *H*_*q*_*s*__ = 0, *H*_*q*_1__ = 1, *H*_*q*_2__ = 2, and *H*_*q*_3__ = 3. If *q*_2_ receives the position correction information from *q*_1_, the adjusted position predictions indicate that *q*_1_ will lose contact with *q*_2_. Since *H*_*q*_1__is the minimum *H* stored in *q*_2_, after *q*_2_ node deletes the *q*_1_, it modifies its own *H* value to ∞ and triggers an extra position correction broadcast. Also, when *q*_3_ receives the position correction by this broadcast, it continues the same operation.

In the communication process, the effectiveness of link should be guaranteed from the sending of *p*'s request to *q*_*s*_'s response return to *p*. Once a pair of nodes on the link are disconnected, it should be processed correspondently. The relatively ideal way is to find the third node before disconnection and to maintain the link. Another simple method is to notify *p* and *q*_*s*_ to abandon the appointment on both ends. Thus, it can avoid feedback loss which will result in information consistency. Then the *p* initiates the request process again.

As the vehicle's speed is fast and unstable, the time of link maintains a relatively short time. The frequently broken links will lead to repeated reconnection. To avoid this, higher reliable links are required. So in the *q*'s neighbor nodes state table, the reliability attribute is added for each neighbor node. The reliability refers to the prediction accuracy at *t* + *t*_*f*_ and the help to effective datagram transmission on the link. It mainly depends on the following two conditions: (1) during *t* to *t* + *t*_*f*_ period, the relative position between *q*′ and *q*. If the distance is far, on the one hand, the weak signal can lead to transmission instability; on the other hand, since *q*′ has been close to the edge of the *q* communication range, the slight change of the vehicles will make *q*′ out of *q*'s communication range and cause broken connection. If *q*′ and *q* are close, the data transmission will take place in a short distance. The datagram will not move a lot in geographical location; thus, it influences the efficiency of data transmission. Thus supposing *U*_*q*,*q*′_(*t*′) = 2 | *D*_*q*,*q*′_(*t*′) − *R* | /*R* refers to the transmission reliability of *q*, and *q'*

(1) Uq,q′(t,t+tf)=integration(t,t+tf).

Modify the condition of correcting *H*_*q*_ in Section 2.1. Given the reliable threshold *r* ∈ [0,1] and after *q*_1_ node receives the broadcast of *q*_2_ node, if state (*q*_2_) = active, *H*_*q*_2__ + 1 < *H*_*q*_1__, and *U*_*q*,*q*′_(*t*, *t* + *t*_*f*_) > *r*, then modify the *H*_*q*_1__ value to be *q*_2_ + 1.

## 3. Protocol Implementation

In order to further introduce the implementation of the protocol, the pseudocode algorithms of taxi *q* node and passenger *p* node are given, respectively, in this section. To be simple, based on the discussion in the second section, the following several types of datagram in the taxi-call network will be referred to.

Broadcast (*L*, *V*, *H*, *t*_*c*_) represents position broadcast. The taxi node sends periodically, and the nearby taxi node receives.

*M*_1−6_ represents the six kinds of datagrams in the two-end handshaking protocol after the passengers make a request.

*M*_*e*_ represents the cancelation of the reserved datagram while finding the abnormal disconnection.

Algorithm 1 is divided into two parts. The timer gets the position and speed information for a short time (e.g., 3 seconds). If the interval exceeds *t*_*f*_, then send broadcast to the nodes around. According to the current measured data, the position at *t* + *t*_*f*_ can be predicted. If there exists significant difference between the position and the position of the last broadcast, then send broadcast without considering whether the time intervals exceed *t*_*f*_ or not. The timer will also inspect the neighbor node that is not updated over time in the adjacency list and set these nodes as inactive. Since the time in the record is the position measuring time, to consider the communication delay, the overtime window will be slightly amplified to 1.1*t*_*f*_. If not updated for more than two broadcast cycles, then delete the node.

As for the inactive nodes, check whether they are in the current communication connection. If it is true, then send *M*_*e*_ to other connected nodes to cancel the conformation.

The other part of Algorithm 1 is the listener. After the listener receives a datagram, he/she judges data types. If it is the position broadcast datagram, then update the list. Once the predicted node position changes are identified in the current connection and in a short time will overpass the scope of communication, and then notify other nodes on the connection to cancel the appointment. When accepting other types of datagram, again transmit or respond based on the protocol.

Algorithm 2 mainly includes two timers and a listener. When initiating the request, timer_1_ is set. At the same time, the listener will put the received response to the response queue. When timer_1_ is triggered, it checks the queue. If the queue is empty, it means no response, and it sends the request again. Otherwise, it chooses the response that has the nearest transmission distance from passengers to not-taken taxis in order to make appointment. And it simultaneously sends veto to other response nodes. After sending the appointment datagram, set timer_2_. Once no appointment conformation *M*_4_ in a certain time *M*_4_, then the timer_2_ will be launched, and send request *M*_1_ again. Attention here, once the listener receives an *M*_*e*_, the datagram cancellation is caused by connection error. Then immediately start the timer_2_ and send request again.

## 4. Performance Analysis

In order to evaluate the performance of the proposed routing protocol, the paper uses NS2 and VanetMobiSim to simulate.

The NS2's main parameters are shown in Table 1. The simulation scene size is 5 × 5 square kilometers. In VanetMobiSim simulation, the scene size is 5 × 5 square kilometers too. In the total area there are 20 traffic signals and the entire simulation area is divided into several regions. In every region the density of streets and the allowed maximum speed are different. The regions which have higher street density have lower allowed maximum speed. When vehicles get into the corresponding region, they choose the speed from street allowed maximum speed and self-allowed maximum speed with a low one. In all of our simulations, the empty taxis account for 40%.

We set the interval of route maintain message *T*_*b*_ to be equal to 10 s and then set the maximum speed of taxi nodes to be equal to 60 km/h. When we adjusted the density of taxi nodes, we got the probability of passenger node *P* to find empty taxi node *T*_*D*_ and the probability of node *P* to receive the message from *T*_*D*_ as shown in Table 3.

As shown in Table 3, when node density is too low, the transmission range will be so small that it is hard to maintain the information of nodes for a long time. Hence, when *P* appears, the number of nodes that *P* can communicate with is small and the time of link is short so that it is hard to communicate with *T*_*D*_. As the node density becomes higher, situations are getting better, but some nodes have higher velocity, which leads to more failure. Because the links chosen are the links that have long duration, so the probability of node *P* receiving acknowledgement messages from node *T*_*D*_ is pretty high.

Then if we fix the density of taxi nodes as 5 per km^2^ and fix the transmission range as 500 m, we will get the relation of the maximum speed and the successful ratio.

**Figure 3:**
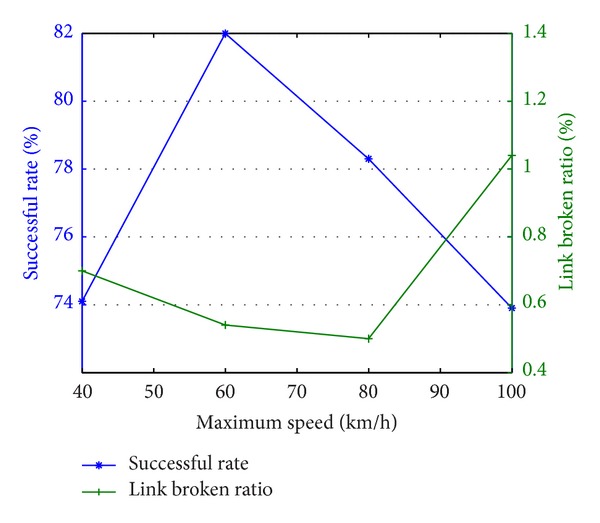
The relation of the maximum speed of taxi nodes and the ratio of passenger node contacting empty taxi nodes successfully.

We called it successful calling when the passenger node finds the empty taxi node. In Figure 3, we could see that when the maximum speed of taxi is equal 60 km/h, it has the highest successful ratio which enables the passenger node to find the empty taxi nodes. This is because when the node's moving speed is low, the node can contact with fewer other nodes. And when the node's moving speed is high, the network topology changes fast, which causes fewer available routing link and thus causes lower successful calling.

**Figure 4:**
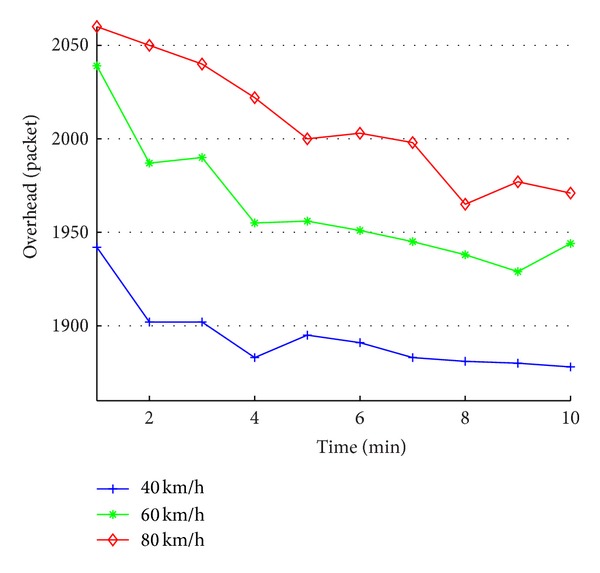
The impact of node's moving speed on the amount of switching packets of entire network.

Figure 4 shows the impact of node's moving speed on the amount of switching packets of entire network.

As shown in Figure 4, the amount of switching packets grows up as the vehicle movement speed increases. This is because when the speed of node movement increases, it causes more correcting message, resulting in the increase of network data traffic. In addition, in one simulation, the routing overhead is higher at the beginning and gradually declines over time. This is because when the simulation starts, all taxi nodes are active at the same time and all taxi nodes broadcast at the same time, which causes more channel collision, leading to an increasing number of retransmissions. As time progresses, due to the correcting message, the taxi routing maintenance cycles gradually stagger and the entire network routing overhead declines.

**Figure 5:**
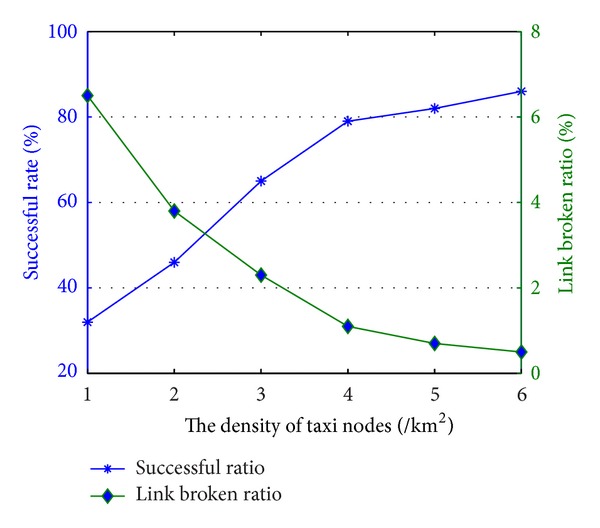
The impact of nodes density on successful ratio.

Then we adjust the density of taxi nodes in the region. The average density of taxi nodes is set at 1–6 per square kilometer and the vehicle maximum speed is set at 60 km/h and then the results are shown in Figure 5.

As shown in Figure 5, when the taxi nodes are scarce, the rate of the success calling drastically reduces. While increasing the number of taxi nodes, the rate of success calling improves accordingly. This is because when the number of taxi nodes is too small, there is no taxi node around passenger node so that the passenger node cannot contact with any node, resulting in more calling failure. When the number of taxi nodes increase and, the passenger node sends an appointment request, the number of alternative routing paths increases, so the node can choose a more stable routing path to delivery packet; thereby the rate of success calling will increase.

At last, we control node velocity between 0 and 20 m/s; interval of *T*_*b*_ 10 s, transmission range is 500 m, and the density is 4 per square kilometers. Compared to AODV (show as Table 4), the results are shown in Table 2. We can see that the proposed routing protocol performs better than AODV. More importantly, our protocol communication data is scattered throughout the network, while AODV is relatively concentrated. As a result, our protocol performs better in the channel collision. It is because AODV requires a great deal of broadcast to maintain the entire link routing information and has no efficient methods to deal with the disconnection of links.

## 5. Conclusion

This paper proposes a routing protocol for taxi-calling application. Firstly, it analyzes scenes of taxi-calling application and studies the differences between the common vehicle application scene and the taxi-calling application scene. Secondly, it proposes the routing information maintaining algorithm based on position prediction which performs better reliability. Thirdly, a self-network forming method for taxi-calling application is drawn up.

Our protocol queries the destination node by nodes' keeping the information of the hop count to destination and decreases the routing cost. Meanwhile, the validity of routes is guaranteed by predicting the future position of themselves and their neighbors.

This protocol has also been evaluated by a traffic simulator. The results indicate that our protocol can efficiently find the target nodes in the area. It has also been found that the number of broadcasts is affected by nodes' velocity. And the fact that our protocol generates 30% less messages than AODV has been proven here.

As to future work, our protocol can probably be extended to solve the low delivery rate problem when the density of vehicle is low.

## Figures and Tables

**Algorithm 1:**
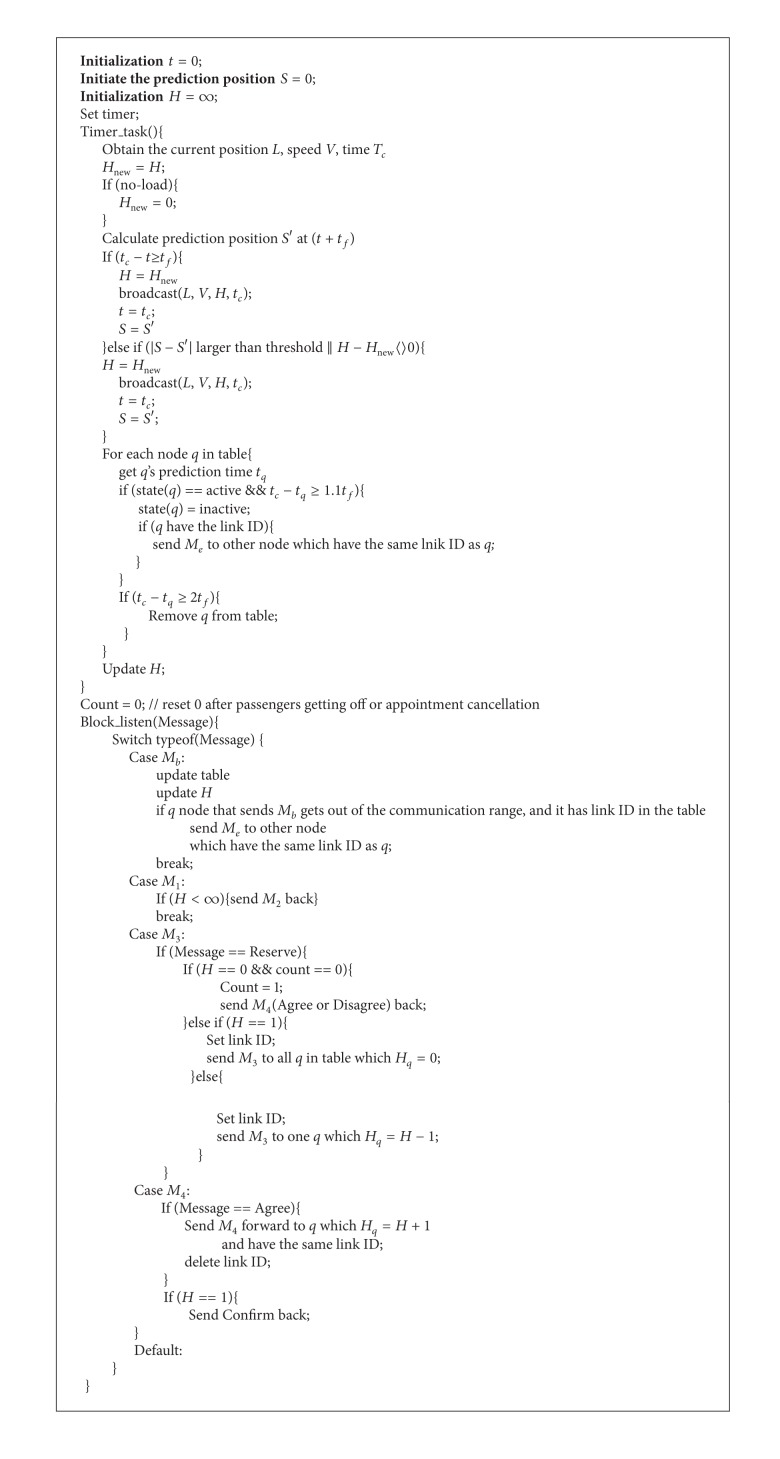
Algorithm of taxi node.

**Algorithm 2:**
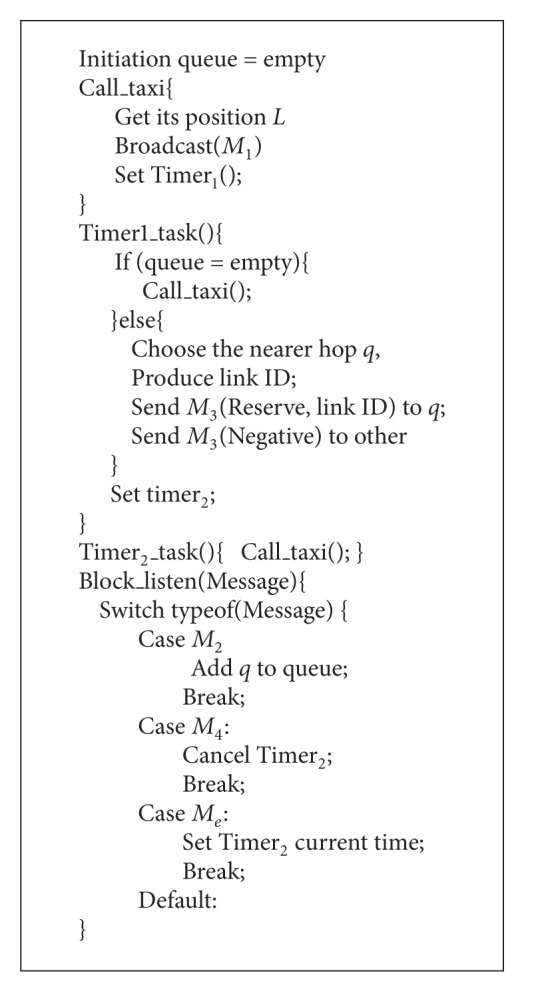
Algorithm of passenger node.

**Table 1:** State of the *q*_1_'s Neighbor Nodes.

Neighbor	*H*_*q*_*n*_,*q*_*s*__	Position	Direction of speed	Link ID	Reliability	Measuring time	State
*q*_2_	3	120.213476E: 30.321768N	*d*_*q*_2__	182378627	0.94	13:11:12′36	Active
*q*_3_	5	120.213484E: 30.321775N	*d*_*q*_3__	452766732	0.83	13:10:37′34	Inactive

**Table 2:** The NS2 parameters.

Parameters	Values
Loss model	Propagation/TwoRayGround
Physical model	Phy/WirelessPhyExt
Antenna	Antenna/OmniAntenna
Transmission rate	2 Mbps
Network protocol	IEEE 802.11

**Table 3:** The impact of nodes density and transmission range on successful calling ratio.

Transmission range	Density of taxi			
	4		6	
	The probability to find *T*_*D*_	The probability to receive from *T*_*D*_	The probability to find *T*_*D*_	The probability to receive from *T*_*D*_
200 m	14.8%	78.5%	49.8%	95.5%
300 m	44.3%	91.2%	78.4%	94.2%
500 m	61.1%	94.1%	87.3%	94.6%

**Table 4:** Compare with AODV.

	CBLIGR	AODV
Delivery rate/(%)	61.1	52.3
Control message/packet	1206.8	1815.0
